# Increased inspiratory resistance affects the dynamic relationship between blood pressure changes and subarachnoid space width oscillations

**DOI:** 10.1371/journal.pone.0179503

**Published:** 2017-06-27

**Authors:** Magdalena Wszedybyl-Winklewska, Jacek Wolf, Ewa Swierblewska, Katarzyna Kunicka, Kamila Mazur, Marcin Gruszecki, Pawel J. Winklewski, Andrzej F. Frydrychowski, Leszek Bieniaszewski, Krzysztof Narkiewicz

**Affiliations:** 1 Institute of Human Physiology, Medical University of Gdansk, Gdansk, Poland; 2 Department of Hypertension and Diabetology, Medical University of Gdansk, Gdansk, Poland; 3 Department of Cardiovascular Diseases, International Clinical Research Center, St. Anne’s University Hospital in Brno (FNUSA), Brno, Czech Republic; 4 Department of Biomedical Engineering, Faculty of Electronics, Telecommunications and Informatics, Gdansk University of Technology, Gdansk, Poland; 5 Department of Radiology Informatics and Statistics, Medical University of Gdansk, Gdansk, Poland; 6 Institute of Health Sciences, Pomeranian University of Slupsk, Slupsk, Poland; 7 Centre for Medical Simulation, Medical University of Gdansk, Gdansk, Poland; university of cambridge, UNITED KINGDOM

## Abstract

**Background and objective:**

Respiration is known to affect cerebrospinal fluid (CSF) movement. We hypothesised that increased inspiratory resistance would affect the dynamic relationship between blood pressure (BP) changes and subarachnoid space width (SAS) oscillations.

**Methods:**

Experiments were performed in a group of 20 healthy volunteers undergoing controlled intermittent Mueller Manoeuvres (the key characteristic of the procedure is that a studied person is subjected to a controlled, increased inspiratory resistance which results in marked potentiation of the intrathoracic negative pressure). BP and heart rate (HR) were measured using continuous finger-pulse photoplethysmography; oxyhaemoglobin saturation with an ear-clip sensor; end-tidal CO_2_ with a gas analyser; cerebral blood flow velocity (CBFV), pulsatility and resistive indices with Doppler ultrasound. Changes in SAS were recorded with a new method i.e. near-infrared transillumination/backscattering sounding. Wavelet transform analysis was used to assess the BP and SAS oscillations coupling.

**Results:**

Initiating Mueller manoeuvres evoked cardiac SAS component decline (-17.8%, P<0.001), systolic BP, diastolic BP and HR increase (+6.3%, P<0.001; 6.7%, P<0.001 and +2.3%, P<0.05, respectively). By the end of Mueller manoeuvres, cardiac SAS component and HR did not change (+2.3% and 0.0%, respectively; both not statistically significant), but systolic and diastolic BP was elevated (+12.6% and +8.9%, respectively; both P<0.001). With reference to baseline values there was an evident decrease in wavelet coherence between BP and SAS oscillations at cardiac frequency in the first half of the Mueller manoeuvres (-32.3%, P<0.05 for left hemisphere and -46.0%, P<0.01 for right hemisphere) which was followed by subsequent normalization at end of the procedure (+3.1% for left hemisphere and +23.1% for right hemisphere; both not statistically significant).

**Conclusions:**

Increased inspiratory resistance is associated with swings in the cardiac contribution to the dynamic relationship between BP and SAS oscillations. Impaired cardiac performance reported in Mueller manoeuvres may influence the pattern of cerebrospinal fluid pulsatility.

## Introduction

Spontaneous respiration is known to modulate heart-driven cardiovascular oscillations. Left ventricle (LV) stroke volume reduction characteristic for normal inspiration can be partially explained by a diminished cardiac filling evoked by heart rate acceleration. Respiration synchronous changes in stroke volume are also caused by variation in intrathoracic pressure and direct mechanical interactions between the right (RV) and the left ventricles. Animal data demonstrates that exaggerated intrathoracic pressure evoked by Mueller manoeuvres augments both venous blood return to the RV and afterload for the LV resulting in fall in LV stroke volume [1,2,3]. These findings from sixties and seventies of the last century have been recently confirmed in human. Negative thoracic pressure evoked by Mueller manoeuvres increases the right-to-left pressure gradient across the atrial septum [4] changes myocardial mechanics [5], decreases LV stroke volume, LV ejection fraction and cardiac output [6].

Heart cycle and respiration are the main drivers affecting cerebrospinal fluid (CSF) movement. Deep inspiration results in substantial increase in CSF shift from cranial compartment to the cervical spinal canal [7]. Breathing-driven CSF dynamics and bidirectional motion through the basal cisterns and intraventricular passageways, including CSF movements present in subarachnoid spaces (SAS), have been recently described in humans [8]. CSF flow evoked by thoracic pressure reduction during inspiration is transmitted to the SAS via the interconnected venous plexus around the thoracic spinal column and within the spinal canal. In addition, diminished thoracic pressure directly affects the hydrostatic pressure that drives the low-resistance paravenous, venous, and lymphatic CSF drainage [9].

The width of SAS can be measured at real time using a novel method based on infrared radiation called near-infrared transillumination/backscattering sounding (NIR-T/BSS). The rationale for NIR-T/BSS methodology is that the CSF within the SAS acts as a propagation duct for infrared radiation [10,11].

In our previous study with NIR-T/BSS we documented rapid SAS oscillations associated with systolic-diastolic changes in blood volume of cerebral circulation [12]. Subsequent analysis of wavelet phase and amplitude coherences identified similarity of these two oscillators, and allowed us to clearly identify a proportion of systemic blood pressure (BP) oscillations with corresponding SAS changes. Coupling functions represent a powerful methodology to unveil the neurophysiological mechanisms governing organism response to several challenges. Therefore in the series of our recent studies we investigated the effect of apnoea [12,13], sympathetic activation [14] and hypoxia [15]—the stimuli typically seen in obstructive sleep apnoea (OSA). In particular we demonstrated that BP and SAS amplitudes coherence diminish during apnoea in normal healthy subjects [12] which is not the case in professional apnoea divers [13]. Sympathetic nervous system seems rather to stabilise the system [13,14]. On the contrary, hypoxia declines BP and SAS amplitudes coherence [15].

Taken together, increased respiratory resistance substantially affects cardiac performance and disturbs CSF motion. In addition, high sympathetic drive caused by hypoxia and breathing difficulty augments BP [16,17]. In this study we aimed at assessing the effect of increased inspiratory resistance on the coupling between BP and SAS oscillation signals. The high sampling frequency (70 Hz) of NIR-T/BSS allows for signal analysis up to 35 Hz according to Nyquist theorem. We decided to analyse collected signals up to 10 Hz due to lack any physiological process over this frequency. The power spectrum density of SAS oscillations shows clear peaks at the cardiac frequency (f0) and its harmonics (f1, f2, f3) [11,12]. We hypothesised that increased inspiratory resistance introduced by series of Mueller manoeuvres would diminish the cardiac contribution to the relationship between BP and SAS oscillations.

## Materials and methods

### Ethical approval

The study conformed to the standards set by the Declaration of Helsinki. The experimental protocol and the study design were approved by the Ethics Committee of the Medical University of Gdansk (NKEBN/48/2011). All subjects gave written informed consent to participate in the study.

### Subjects

Experiments were performed in a group of 20 healthy, non-smoking volunteers (Table 1). Nicotine, coffee, tea, cocoa and methylxanthine-containing food and beverages were not permitted for 8 hours before the tests. Additionally, prior to each test, the volunteers were asked to rest comfortably for 30 minutes in the supine position.

**Table 1:** Characteristics of the study participants. Data are presented as mean values and standard deviations (SD).

	Males (n = 9)	Females (n = 11)
Age (years)	26.3 ± 8.4	20.1 ± 2.0
BMI (kg*m^-2^)	23.2 ± 4.2	22.0 ± 3.5
cc-TQ (AU)	83.0 ± 34.9	60.8 ± 35.4
sas-TQ (AU)	130.8 ± 61.1	111.9 ± 87.0
SBP (mmHg)	125.7 ± 4.9	117.3 ± 11.4
DBP (mmHg)	74.2 ± 6.3	70.1 ± 3.9
HR (beats*s^-1^)	70.7 ± 9.6	78.6 ± 10.8
CBFV (cm*s^-1^)	41.3 ± 7.8	42.7 ± 9.4
RI	0.67 ± 0.03	0.61 ± 0.11
PI	1.48 ± 0.19	1.25 ± 0.38
SaO_2_	98.3 ± 1.1	98.6 ± 1.3

cc-TQ–cardiac component of the subarachnoid width (heart-generated pial artery pulsation, from 0.5 Hz to 5.0 Hz); sas-TQ–slow component of the subarachnoid width (<0.05 Hz); SBP–systolic blood pressure; DBP–diastolic blood pressure; HR–heart rate; CBFV–cerebral blood flow velocity; PI–pulsatility index; RI–resistive index; SaO2—oxyhemoglobin saturation; kg–kilograms; m- meters; AU–arbitrary units; mmHg—millimeters of mercury; s–seconds

### Experimental design

**Fig 1:**
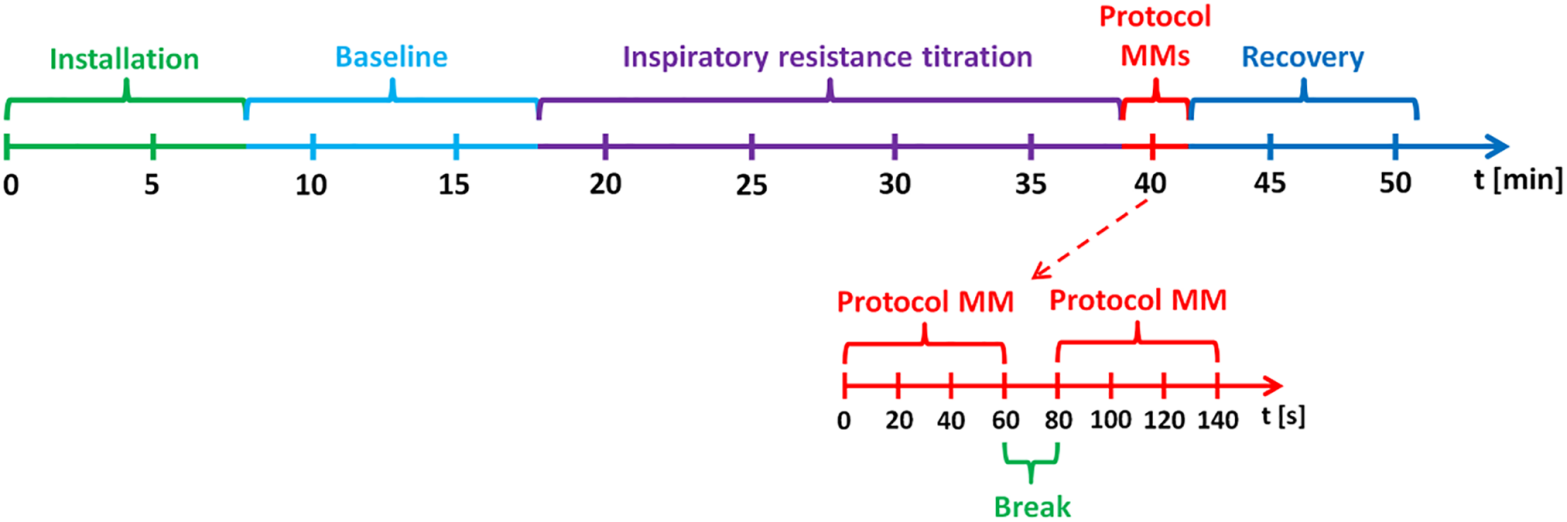
Schematic representation of the study design.

After baseline measurements had been obtained, participants were subjected to controlled repetitive Mueller manoeuvres. The procedure was performed with a modified single-use Threshold IMT® (Phillips-Respironics, Best, the Netherlands) device, primarily designed for rehabilitation of the inspiratory muscles. The device consists of a mouth-piece, container and a calibrated adjustable valve which allows for precise control of the inspiratory resistance ranging from -2 to -40 cm H_2_O. The target pressure of a single experimental Mueller manoeuvres equalled -40 cm H_2_O, which was applied six times in a row over the period of one minute. The repetitive Mueller manoeuvres were then followed by 20 s of undisturbed recovery breathing, and looped for 2 times The idea behind this protocol was to investigate the Mueller manoeuvres which closely mimic repetitive hypopneas commonly seen in real-life in human obstructive sleep apnoea (OSA) syndrome. Since the target Mueller manoeuvres described by the study protocol are rather forceful procedures, subjects were first titrated with inspiratory resistance as well as time length of its application. Throughout the first 20 minutes of the experiment, the inspiratory resistance was gradually enhanced starting from -2 cm H_2_O and was applied in two to three repetitions. The titration procedure allowed the subjects to adapt to the experiment which ensured repeatable signalling. Averaged data obtained from a two-minute series of experimental Mueller manoeuvres were analysed. Graphical presentation of study design is shown in Fig 1.

For wavelet transform analysis, 10 s data windows were taken from baseline and from the last one-minute series of Mueller manoeuvres. From the Mueller manoeuvres series, 10 s data windows corresponding to minimum and maximum wavelet coherence (WCO) were taken.

### Measurements

ECG was recorded using a standard electrocardiograph. Systolic (SBP) and diastolic blood pressure (DBP), along with HR, were measured using continuous finger-pulse photoplethysmography (Finometer, Finapres Medical Systems, Arnhem, the Netherlands). Finger blood pressure was calibrated against brachial arterial pressure. Oxyhaemoglobin saturation (SaO_2_) was measured continuously (Massimo Oximeter, Massimo, Milan, Italy) with an ear-clip sensor. Expired air was sampled from the mouthpiece and the end-tidal CO_2_ (EtCO_2_) was measured using a gas analyser (PNT Digital M.E.C. Group, Brussels, Belgium). Doppler ultrasound of the internal carotid artery was performed (*Vivid 7*, GE Healthcare; Little Chalfont, UK) to assess the mean cerebral blood flow velocity (CBFV), pulsatility index (PI) and resistive index (RI). The SAS width was recorded separately for right and left hemispheres with the head-mounted sensors of the NIR-T/BSS device (SAS 100 Monitor, NIRTI SA, Wierzbice, Poland). Cardiac (0.5–2.0 Hz) and slow (<0.5 Hz) SAS components were calculated by the SAS Monitor software based on empirical mode decomposition concept. The theoretical and practical foundations of the NIR-T/BSS method were described in earlier studies [10,11]. BP and SAS pulsation signals were acquired at 70 Hz via a data acquisition unit (Powerlab/16SP ML795, AD Instruments). All variables were recorded continuously or videotaped, and the signals were routed to a hard drive for further analyses.

### Wavelet analysis

Wavelet analysis transform a time signal from the time domain to the time-frequency domain [18,19]. We used this method in our earlier work [12] as a tool to characterize changes in SAS and BP. To find relationship between signals firstly we calculated a wavelet coherence (WCO) [12,20]. A value of 1 (0) indicates high (low) correlation between two time series. Additionally, we estimated a wavelet phase coherence (WPCO) which identifies possible relationships by evaluating the match between the instantaneous phases of two signals [12,19]. The value of the phase coherence is between 0 and 1. This value quantifies the tendency of the phase difference between the two signals to remain constant at a particular frequency [19]. When two oscillations are unrelated, their phase difference changes with time.

### Statistical analysis

We used the Wilcoxon signed rank test to compare the changes in WCO, WPCO, the width of subarachnoid space, pial artery pulsation, SBP, DBP, HR, CBFV, RI, PI, SaO_2_ and EtCO_2_ in response to the Mueller manoeuvres series. Additionally, we performed a multivariate linear regression analysis to identify which variables influenced the changes in the width of subarachnoid space and pial artery pulsation in response to the Mueller manoeuvres series. The sample size was determined on the basis of test power analysis (20 individuals had a power greater than 80% to detect the assumed SAS changes).

WCO and WPCO assessment was performed with all-time series and generated corresponding surrogate time series. To generated surrogates we used iterative amplitude adjusted Fourier transform–IAAFT [21]. Original WCO and WPCO located above the surrogate WCO and WPCO are considered statistically significant.

## Results

Increased resistance evoked by Mueller manoeuvres resulted in an evident haemodynamic response consisting of two phases.

In the beginning (1^st^ phase) of Mueller manoeuvres BP rise was associated with diminished amplitude of SAS cardiac component. HR was slightly increased, PI and RI did not change.

By the end of Mueller manoeuvres (2^nd^ phase), with slight but constant SaO_2_ decline, cardiac SAS component and HR returned to baseline values while BP remained elevated. Subsequently, CBFV increased, representing a typical response to hypoxia. PI and RI diminished.

Importantly, slow SAS component and EtCO_2_ did not change throughout the Mueller manoeuvres. The summary of results is presented in Table 2.

**Table 2:** Effects of 60 s Mueller manoeuvres series on SAS, SBP, DBP, HR, CBFV, PI, RI and SaO_2_. Data are presented as mean values and standard deviations (SD). All % changes are calculated with reference to baseline values.

	Baseline	Beginning of MMs (13 ± 5 s)	Beginning of MMs vs. Baseline (%)	End of MMs (40 ± 5 s)	End of MMs vs. Baseline (%)
cc-TQ (AU)	73.2 ± 59.4	60.2 ± 45.6^***^	82.2	74.9 ± 47.6^NS^	102.3
sas-TQ (AU)	117.8 ± 94.6	128.5 ± 94.7^NS^	109.1	125.0 ± 89.9^NS^	106.1
SBP (mmHg)	127.9 ± 7.3	136.0 ± 10.7^***^	106.3	144.0 ± 12.7^***^	112.6
DBP (mmHg)	77.7 ± 7.9	82.9 ± 8.2^***^	106.7	84.6 ± 10.7^***^	108.9
HR (beats*sec^-1^)	75.0 ± 9.0	77.0 ± 11.0^*^	102.7	75.0 ± 9.1^NS^	100.0
CBFV (cm*sec^-1^)	42.1 ± 9.7	43.2 ± 10.4^NS^	102.6	48.0 ± 9.8^***^	114.0
RI	0.62 ± 0.08	0.61 ± 0.09^NS^	98.4	0.55 ± 0.10^**^	88.7
PI	1.31 ± 0.36	1.27 ± 0.34^NS^	96.9	1.12 ± 0.37^**^	85.5
SaO_2_	98.8 ± 0.6	98.6 ± 0.7^***^	99.8	97.0 ± 1.5^***^	98.2
EtCO_2_	35.3 ± 5.7			35.7 ± 5.9	101.1

*P<0.05

**P<0.01

***P<0.001

cc-TQ–cardiac component of the subarachnoid width (heart-generated pial artery pulsation, from 0.5 Hz to 5.0 Hz); sas-TQ–slow component of the subarachnoid width (<0.05 Hz); SBP–systolic blood pressure; DBP–diastolic blood pressure; EtCO_2_—end-tidal CO_2_; HR–heart rate; MBP–mean blood pressure; CBFV–cerebral blood flow velocity; PI–pulsatility index; RI–resistive index; SaO2—oxyhemoglobin saturation; AU–arbitrary units; mm Hg—millimeters of mercury; s–seconds

Multivariate regression analysis revealed relation between change in cardiac SAS component and BP in the beginning and by the end of Mueller manoeuvres (Table 3).

**Table 3:** Multivariate regression analysis. Model explaining changes in cardiac SAS component with relation to cerebral blood flow velocity, heart rate, oxyhaemoglobin saturation and blood pressure fluctuations in response to the beginning and terminal part of the Mueller manoeuvres.

	(β)*	SD	P
13 ± 5 s of Mueller manoeuvres			
delta CBFV	0.043	0.84	0.80
delta HR	-0.090	0.76	0.59
delta SaO2	-0.115	0.76	0.49
delta SBP	0.367	0.72	0.02
40 ± 5 s of Mueller manoeuvres			
delta CBFV	0.028	0.72	0.09
delta HR	0.077	0.76	0.65
delta SaO2	-0.261	0.71	0.11
delta SBP	0.334	0.71	0.04

CBFV–cerebral blood flow velocity, HR–heart rate; SaO2 –blood oxygen saturation; SBP–systolic blood pressure (continuous recordings); SD–standard deviation.

* standardized slope in the same units of measure

**Fig 2:**
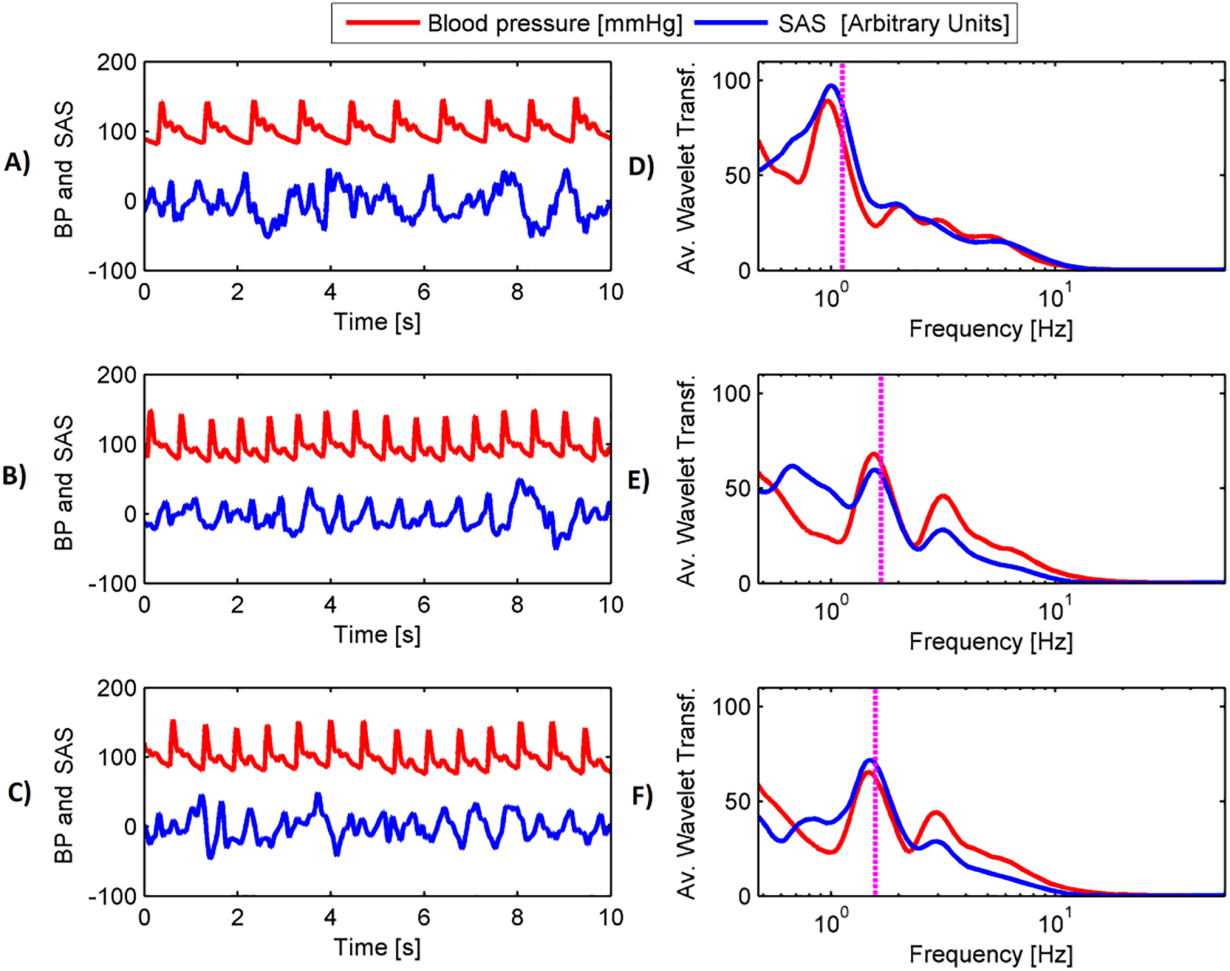
Representative tracings from 10 s BP and SAS signals with corresponding wavelet transforms; baseline (panel A), WCO minimum (panel B) and WCO maximum (panel C) from one of participants. The SAS signal (in blue) is less regular than the BP signal (in red). Wavelet transform analysis reveals BP and SAS peaks at cardiac frequency marked with vertical magenta dotted lines (SAS analysis in blue, BP in red). Alterations in cardiac frequency are apparent throughout the Mueller manoeuvres.

The results of wavelet transform analysis of the 10 s BP and SAS signals is shown in Fig 2. Alternating BP and SAS peaks at the human cardiac frequency is visible.

**Fig 3:**
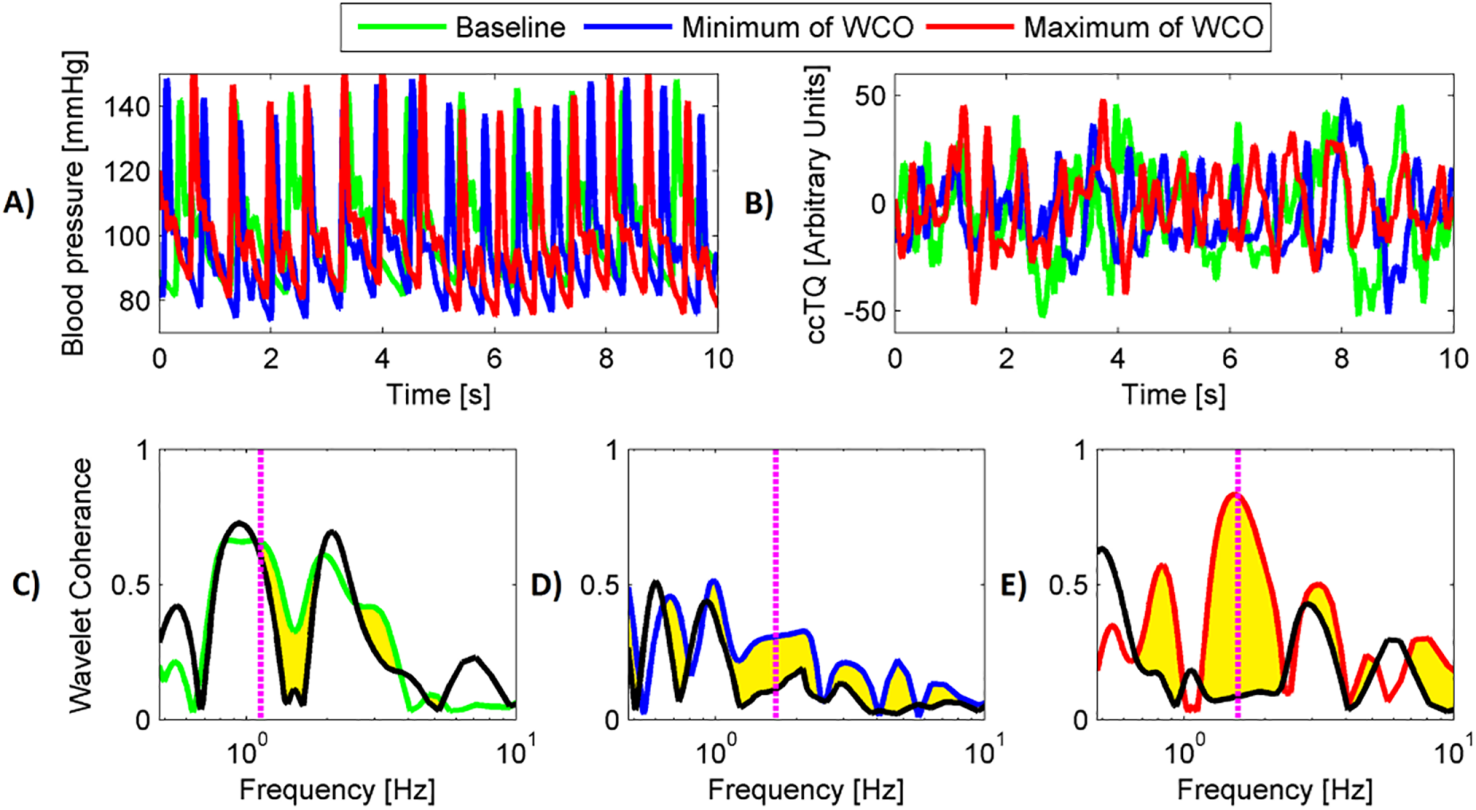
Representative tracings of 10 s signals of baseline (in green), 10 s of minimum WCO (in blue) and 10 s of maximum WCO (in red). BP oscillations (panel A), SAS oscillations (panel B) and WCO (panels C, D and E). Cardiac frequency is indicated by vertical, magenta, dotted lines. Black solid lines (panels from C to E) illustrate results obtained for IAAFT surrogates.

**Fig 4:**
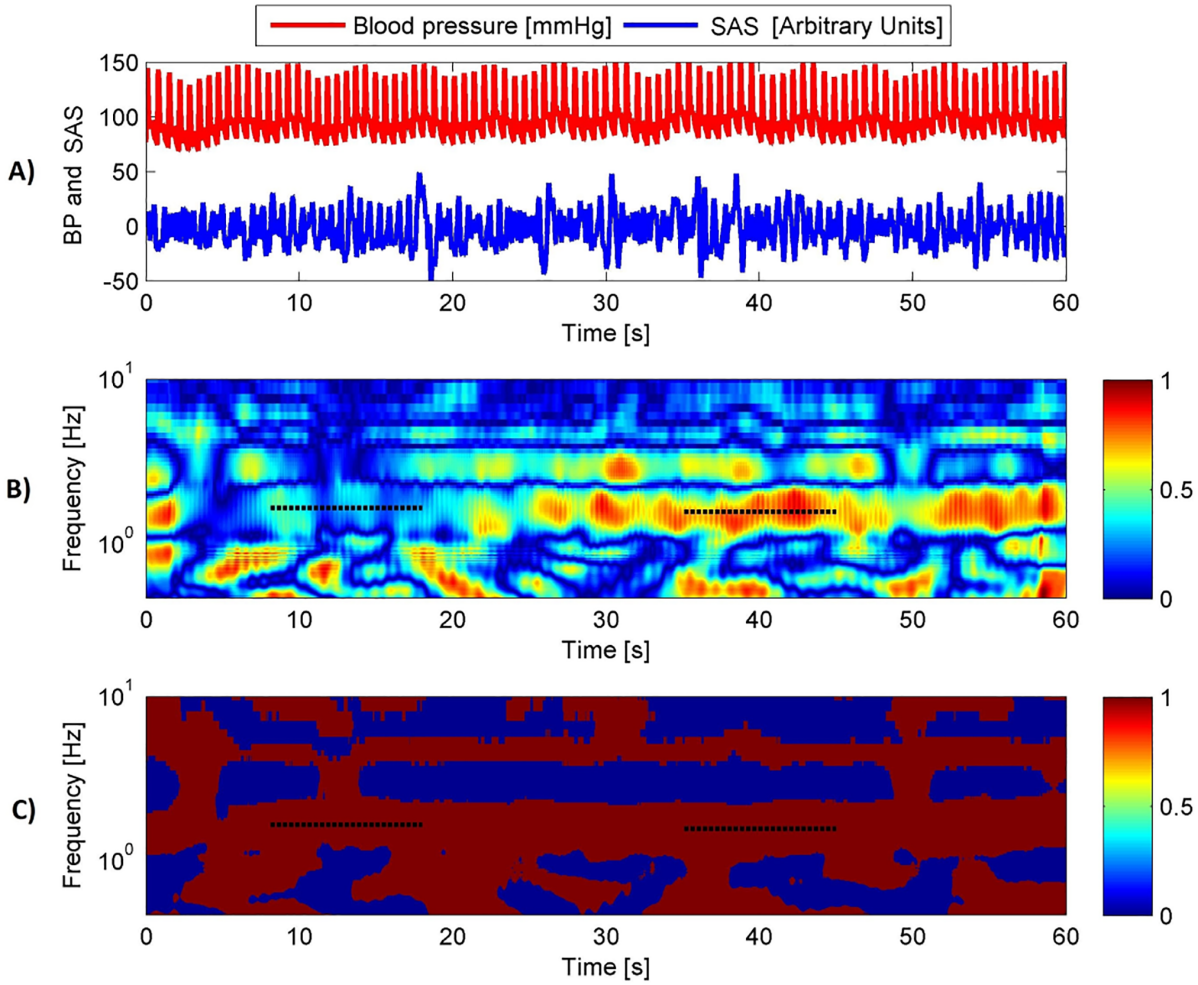
Representative WCO (panel B) and WPCO (panel C) tracings. BP (red) and SAS (blue) signals are provided in the panel A. WCO reaches its minimum between 8 and 18 s of the Mueller manoeuvres, and later upon recovery, reaches its maximum between 35 and 45 s for cardiac frequency. WPCO remains stable. Cardiac frequency is indicated by horizontal dotted lines.

A significant decrease (about 2.2 times for the right hemisphere) in wavelet coherence (WCO) found in the first half of the Mueller manoeuvres series (between 8 and 18 s) was followed by recovery by the end of Mueller manoeuvres (between 35 and 45 s, Table 4, Figs 3 and 4). Wavelet phase coherence (WPCO) was relatively high at baseline and did not change throughout the Mueller manoeuvres. The WCO swings during Mueller manoeuvres are shown in Fig 4. Taking together, BP and SAS signals amplitudes coupling decreases in response to acute reduction of intrathoracic pressure and recovers after several cycles of breathing against increased inspiratory resistance. Phases of both signals are not affected by the procedure.

**Table 4:** Effects of a 60 s Mueller manoeuvres series on WCO and WPCO between BP and SAS oscillations at cardiac frequency. Data are presented as mean values and standard deviations (SD). The minimum and maximum correspond to WCO minimum and maximum values during the Mueller manoeuvres series.

WCO / WPCO	Baseline	Minimum	Minimum vs. Baseline (%)	Maximum	Maximum vs. Baseline (%)	Maximum vs. Minimum (%)
**WCO left WCO right WPCO left WPCO right**	0.65 ± 0.15 0.52 ± 0.18 0.86 ± 0.36 0.87 ± 0.35	0.44 ± 0.30 0.28 ± 0.26 0.85 ± 0.36 0.83 ± 0.35	67.7^*^ 54.0^**^ 98.8^NS^ 95.4^NS^	0.67 ± 0.22 0.64 ± 0.17 0.78 ± 0.42 0.93 ± 0.26	103.1^NS^ 123.1^NS^ 90.7^NS^ 106.9^NS^	152.3^**^ 228.6^***^ 91.8^NS^ 112.0^NS^

*P<0.05

**P<0.01

***P<0.001

WCO–wavelet coherence; WPCO–wavelet phase coherence; cc-TQ–cardiac component of transillumination quotient (pial artery pulsation); left–left hemisphere; right–right hemisphere; SD–standard deviation

All-time series are showed on panels A) and B) of Fig 3. Black solid lines (panels from C to E of Fig 3) illustrate results obtained for IAAFT surrogates. Yellow shaded areas indicate significant coherence (panels C, D and E) [22]. Magenta dashed lines show cardiac frequency. All of them are located in areas of significant coherence. Similar behaviour we observed for higher harmonics.

No significant differences were noted between the left and right hemispheres with respect to the analysed variables. We did not observe any sex-related differences.

## Discussion

The main finding of this study is that increased inspiratory resistance modifies the character of heart-driven interactions between BP and SAS oscillations. Pulsatile CSF motion, together with moderate changes in intracranial pressure, are well-recognized and considered indispensable for proper brain functioning [23]. Abnormal arterial/venous pulsatility termed pulse wave encephalopathy is increasingly recognized as a major risk factor for brain tissue damage [24]. In particular changes in pulse wave characteristic may negatively affect the structural properties of white matter [25].

To investigate BP SAS coupling we used a wavelet transform analysis. The method provides windows of adjustable lengths which results in high resolution at cardiac frequency. For instance the Fourier transform assumes stationary of the signal, i.e. that the frequency content does not change over time. Unfortunately in biomedical signals the frequencies of oscillation change very often due to openness of the system. To solve this problem one might have divided this frequency intervals into a set of intervals and used the short time Fourier transform. The wavelet transform based on the same idea but in the case of the continuous wavelet transform, the number of intervals is no longer finite and the frequency resolution correspondingly changes continuously. The method has already been used by us and many others [12–14,18,19,26].

High negative thoracic pressure is considered a key element of OSA pathophysiology [27]. Mueller manoeuvres are often used to mimic obstructive hypopneas occurring during sleep in OSA subjects [28,29]. High sympathetic drive was found to be neutral to the discussed relationship [13,14]. Therefore, negative thoracic pressure appears to be the predominant factor modulating the dynamic relationship between heart-driven BP and SAS oscillations. Here, we demonstrate for the first time a mechanistic link between the dampening of cardiac function occurring during Mueller manoeuvres [4,5,6] and its transmission into the brain haemodynamic. Described swings in coherence between BP and SAS oscillations signals may impair normal CSF circulation and pulse wave characteristics. Our finding may indicate that intrathoracic pressure modifications during continues positive airway pressure (CPAP) treatment or mechanic ventilation procedures may also affect CSF motion. Further studies are warranted to assess the impact of positive intrathoracic pressure on brain haemodynamic.

In our study we decided to analyse a series of Mueller manoeuvres rather than a single manoeuvre [4,6], and the rationale for this was to better reproduce highly prevalent OSA syndrome. Performing the Mueller manoeuvres in 60 s series followed by a titration procedure, better mimicked increased inspiratory resistance and high sympathetic drive characteristic of OSA, and thus may be compared to our other protocols [12,13,14]. We observed typical BP changes [30,31]. As we have earlier shown the analysis of 10 s series are sufficient to accurately perform wavelet transform analysis [12]. However, using 10 s windows we were not able to analyse Mueller manoeuvres-dependent BP variability. To mitigate this limitation of the presented approach we would like to underscore the priority we gave in our protocol to closely mimic OSA syndrome. Therefore, the study was designed to analyse time series rather than selected data points. BP variability during periods of increased inspiratory resistance may likely represent one of the mechanisms leading to diminished BP SAS amplitudes similarity.

Rapid oscillations in the SAS results from heart-generated changes in systolic-diastolic blood volume in cerebral circulation [23,32]. Therefore, a brief decline in cardiac SAS component in response to BP elevation at the beginning of the Mueller manoeuvres series may represent an active autoregulatory process [13,33,34]. Alternatively, decline in fast (cardiac) SAS component may be related to rapid reduction in intrathoracic pressure and subsequent CSF displacement from cranial compartment. During short lasting respiratory perturbations it is very difficult to differentiate between cardiac and respiratory effects on CSF motion [8]. However, the association between BP and cardiac SAS component changes observed by the end of Mueller manoeuvres seems to indicate that pulsatile pressure and flow directly affect CSF pulsatility.

It has been recently shown that abnormal CSF pulsatility is linked to white matter dysfunction in hypertensive patients [35]. Authors using cine phase magnetic resonance imaging (MRI) to visualise CSF dynamics in the aqueduct of Sylvius also confirmed earlier report by our group that impaired jugular outflow augments CSF pulsatility [36,37]. This study is in line with Chen et al. [8] and Dreha-Kulaczewski et al. [9] MRI reports indicating that respiration is one of the major regulators of human CSF flow. Therefore NIR-T/BSS may provide a low-cost alternative to expensive MRI studies, thus facilitating research on CSF dynamics in human. Recent findings in this field indicate that abnormal CSF dynamic is present in several diseases [35,38,39]. It should be noted that NIR-T/BSS and MRI give equivalent modalities for the SAS change measurements (R = 0.81, P<0.001) [40].

The subjects were allowed to freely exhale, while inhalation was associated with increased resistance. As a consequence, the investigated volunteers slightly hyperventilated. EtCO_2_ did not change throughout the Mueller manoeuvres, resulting in stabilization of the SAS width. The relationship between BP and SAS signals was not disturbed by hyperventilation in our previous study [14]. It is also unlikely that an initial WCO decline can be attributed to diminished SaO_2_. The SaO_2_ decrease was moderate and similar to those observed in maximal breath-hold, where large WCO swings were absent [12,13]. We did not measure intrathoracic pressure and sympathetic activity, but these measurements have been well-described in animals and humans and our data are consistent with previous findings. Finally, the number of subjects enrolled in the study (N = 20) is relatively small, thus the results should be generalized with caution. To mitigate, we would like to underscore that previous NIRT/BSS studies showed highly satisfactory reproducibility and repeatability of the method [10,34]; thus, direct within-individual comparisons are reliable [10,34]. It is however recognized that measurements with the use of infrared light (NIR-T/BSS or previously near-infrared spectroscopy; NIRS) do not provide sufficient reliability to with respect to comparisons between individuals. One of the reasons for such limitation of these methods are differences in skull bone parameters [10,41]. Nevertheless, our study did not aim at between-subject comparisons.

## Conclusions

To summarize, we have shown that increased inspiratory resistance is associated with large swings in the heart-generated dynamic relationship between BP and SAS oscillations in healthy subjects. For the first time, our study revealed that the signal resulting from dumped cardiac performance and/or increased BP variability may directly affect the CSF pulsatility. Our study provides new insights into our understanding of CSF motion in the context of exaggerated negative thoracic pressure.
